# HIV incidence among pregnant and postpartum women in a high prevalence setting

**DOI:** 10.1371/journal.pone.0209782

**Published:** 2018-12-28

**Authors:** Rhoderick Machekano, Appolinaire Tiam, Seble Kassaye, Vincent Tukei, Michelle Gill, Florence Mohai, Masepeli Nchepe, Majoalane Mokone, Janet Barasa, Sesomo Mohale, Mosilinyane Letsie, Laura Guay

**Affiliations:** 1 Elizabeth Glaser Pediatric AIDS Foundation, Washington, DC, United States of America; 2 University of Bergen, Bergen, Norway; 3 Georgetown University, Washington, DC, United States of America; 4 Elizabeth Glaser Pediatric AIDS Foundation, Maseru, Lesotho; 5 Ministry of Health, Maseru, Lesotho; 6 George Washington University, Washington, DC, United States of America; Asociacion Civil Impacta Salud y Educacion, PERU

## Abstract

In sub-Saharan Africa, most women who test HIV negative at the first antenatal care encounter are rarely tested again during pregnancy and postpartum, yet data suggests that pregnancy is associated with increased risk of HIV acquisition compared to non-pregnant women. We describe HIV incidence during pregnancy and postpartum in Lesotho, a high prevalence setting, and factors associated with HIV seroconversion. We enrolled a cohort of HIV negative women presenting at health facilities for antenatal care and followed them through delivery up to 24 months postpartum. Women were repeatedly tested for HIV according to the Lesotho Ministry of Health routine rapid HIV testing guidelines and responded to risk behavior questionnaire every three months. We estimated HIV incidence and associated 95% confidence intervals. We used mixed effects Cox regression models to identify independent factors associated with seroconversion accounting for repeated assessment. The estimated overall HIV incidence rate was 1.58 (95% CI: 1.05–2.28) per 100 person- years. The estimated HIV incidence rate during pregnancy (2.61 per 100 person-years, 95% CI: 1.12–5.14) was almost double the estimated HIV incidence during postpartum (1.36 per 100 person-years, 95% CI: 0.83–2.10). Women’s age (14–24 years compared to 25–45 years), multiple sexual partnerships, urethral discharge and no condoms nor pre-exposure prophylaxis were independently associated with HIV infection. There is an increased need for counseling and support of HIV-uninfected pregnant and breastfeeding women to stay HIV-negative, including provision of pre-exposure prophylaxis during this high-risk period, particularly among adolescent and young women.

## Introduction

Prevention of mother-to-child transmission (PMTCT) programs have been successful at identifying pregnant women infected with HIV when they present for their first antenatal (ANC) visit and initiating preventive measures to limit the transmission of HIV to their infants. However, in many sub-Saharan African countries, women and their partners who test HIV-negative at the first ANC contact are rarely tested again during pregnancy or postpartum despite WHO recommendations and national guidelines that state that repeat HIV testing should be conducted in ANC and postnatal care (PNC) [1], remaining at risk for acquiring HIV infection during their pregnancy and while breastfeeding[2]. Data suggest that pregnancy is associated with an increased risk of HIV acquisition compared to non-pregnant women[3][4]. Physiological changes in women during pregnancy, including immune and hormonal alterations and shifts in the vaginal microbiome, are hypothesized as potential drivers of increased susceptibility to infection[5][6]. A meta-analysis of data from 19 African studies estimated the pooled HIV incidence rate during pregnancy was 4.7 per 100 person-years and 2.9 per 100 person-years during the postpartum period[7]. In addition, acute HIV infection is characterized by high viral load[8], and may increase the risk of HIV transmission to infants[7].

Lesotho is one of the countries with a high burden of HIV infection among women of reproductive age. HIV prevalence in the adult population is estimated at 25%[9]. This estimate is higher among women aged 25–29 years, approximately 37%, and greater than 40% among those aged 30–39 years[9]. Identifying the risk and timing of incident HIV infection during pregnancy and postpartum, together with socio-demographic and behavioral factors associated with HIV acquisition is pivotal for initiating effective targeted preventive interventions. No such evaluation has been previously conducted in Lesotho and this information would guide policy decision-makers on targeting HIV prevention programmatic activities, including pre-exposure prophylaxis (PrEP), to pregnant and postpartum women. In addition, the most recent meta-analysis is based on data from studies conducted between 1997 and 2012. However, HIV incidence rates may have changed due to improved prevention programs and changes in treatment guidelines. More recent estimates can indirectly be used to assess effectiveness of the new treatment guidelines. We therefore estimated HIV incidence during pregnancy and postpartum, and factors associated with HIV acquisition in a cohort of HIV-negative women presenting for antenatal care services at selected health facilities in Lesotho.

## Methods

### Study population and procedures

Between September 2013 and August 2015 we enrolled pregnant women presenting for antenatal care at 13 health facilities in 3 districts of Lesotho into a prospective observational study. All five hospitals from the 3 districts plus 8 health facilities of medium (100–200) or high (>200) antenatal care annual volumes were included in the study. Each pregnant woman with unknown HIV antibody status was tested for HIV per the routine Lesotho MOH protocols for HIV antibody testing using serial rapid tests Determine HIV-1/2 AG/AB and Unigold Recombine HIV-1/2 [10,11]. Lesotho’s guidelines recommended that opt-out HIV testing be offered to pregnant women presenting to ANC or in labor with unknown HIV status; if HIV-negative, they should be retested at 36 weeks gestation if the prior test was performed ≥6 weeks earlier, or if not done prior to delivery then during labor and delivery[12]. HIV-negative women were enrolled into an HIV seroincidence cohort study with scheduled repeat HIV testing at 36 weeks gestation, delivery, and every 3–6 months postpartum until 24 months postpartum.

Baseline socio-demographic characteristics were collected at enrollment. At each visit, the study participant responded to a questionnaire on potential risk factors for HIV infection including sexual partner’s HIV status, use of antiretroviral therapy (ART) by HIV-positive partners, sexual history, sexually transmitted infections and condom use. Women presentation for first ANC visit at 20 weeks or less gestational age were considered early ANC presenters otherwise they were considered late ANC presenters. At the time of the study, ART was recommended for HIV-positive adults with CD4 less than 500 cells/mm^3^ or in WHO stage 3 or 4. Trained study team members administered the questionnaire, entering the responses directly into a web-based database with quality control checks using electronic tablets. HIV test results were captured on paper, and then entered into the database.

### Statistical analysis

We summarized baseline characteristics using frequencies and proportions by whether or not study participants had a follow-up HIV test or not after enrollment. We evaluated if there were differences in baseline characteristics between women who had a follow-up HIV test and women who were not tested for HIV after the initial negative test using chi square tests. Similarly, continuous baseline variables were summarized using medians (Min—Max) and compared across follow-up groups using Wilcoxon ranksum tests.

For each study participant who remained HIV negative throughout the study period, follow-up time was calculated as the difference between date of enrolment into the study and the last date of follow-up. If the participant seroconverted, their date of seroconversion was estimated by the mid-point of the last HIV negative date and the first HIV positive date and their follow-up time was estimated by the difference between enrolment date and estimated HIV seroconversion date. We estimated the overall HIV incidence rate by dividing the number of new HIV infections by the total person-years of follow-up and associated 95% confidence interval, assuming a Poisson distribution. We also estimated the overall HIV incidence stratified by age group (16–19 years, 20–24 years and > = 25 years).

Additionally, we estimated HIV seroincidence during pregnancy and post-delivery separately. Pre-delivery follow-up time was calculated by the difference between enrolment and delivery dates for women who remained HIV-negative. For women who seroconverted before delivery (based on the estimated seroconversion date), their follow-up time was estimated by the difference between enrolment date and the estimated seroconversion date. Post-partum follow-up time was estimated among HIV-negative women at delivery by the difference between delivery date and last HIV negative test date or the difference between delivery and the estimated seroconversion date if the women seroconverted after delivery[13,14]. We then estimated pre-delivery and post-partum HIV incidence rate using the appropriate number of seroconversions and follow-up time for the two periods. We compared pre-delivery and post-partum HIV incidence rates using the incidence rate ratio and associated 95% confidence intervals. We also used Kaplan Meier curves to compare the incidence rates between adolescent (16–19 years), young women (20–24 years) and adult (> = 25 years) women.

We summarized the number and proportion of patients ever reporting each potential risk factor during follow-up. However, since risk factors were repeatedly assessed over time within an individual, we used univariate mixed effects Cox regression to identify factors associated with HIV seroconversion. Factors with a p-value < 0.2 in the univariate models were included in a multivariate mixed effects Cox regression model to identify independent factors associated with seroconversion. We used adjusted hazard rate ratios and associated 95% confidence intervals to quantify the associations. All Cox regression models were stratified by geographic region, thereby assuming different baseline hazards rates for the 3 regions because of different follow-up rates. All statistical analyses were performed using Stata version 15.1 (College Station, TX, USA).

### Protection of human subjects

The study was reviewed and received approval by the Lesotho Ministry of Health Research and Ethics Committee, and the Baylor College of Medicine Children’s Foundation Lesotho Institutional Review Board (IRB) in Lesotho, and the U.S.-based George Washington University Committee on Human Research Institutional Review Board. All women were informed of the study protocol and requirements, and provided written informed consent to participate in the study.

## Results

**Fig 1:**
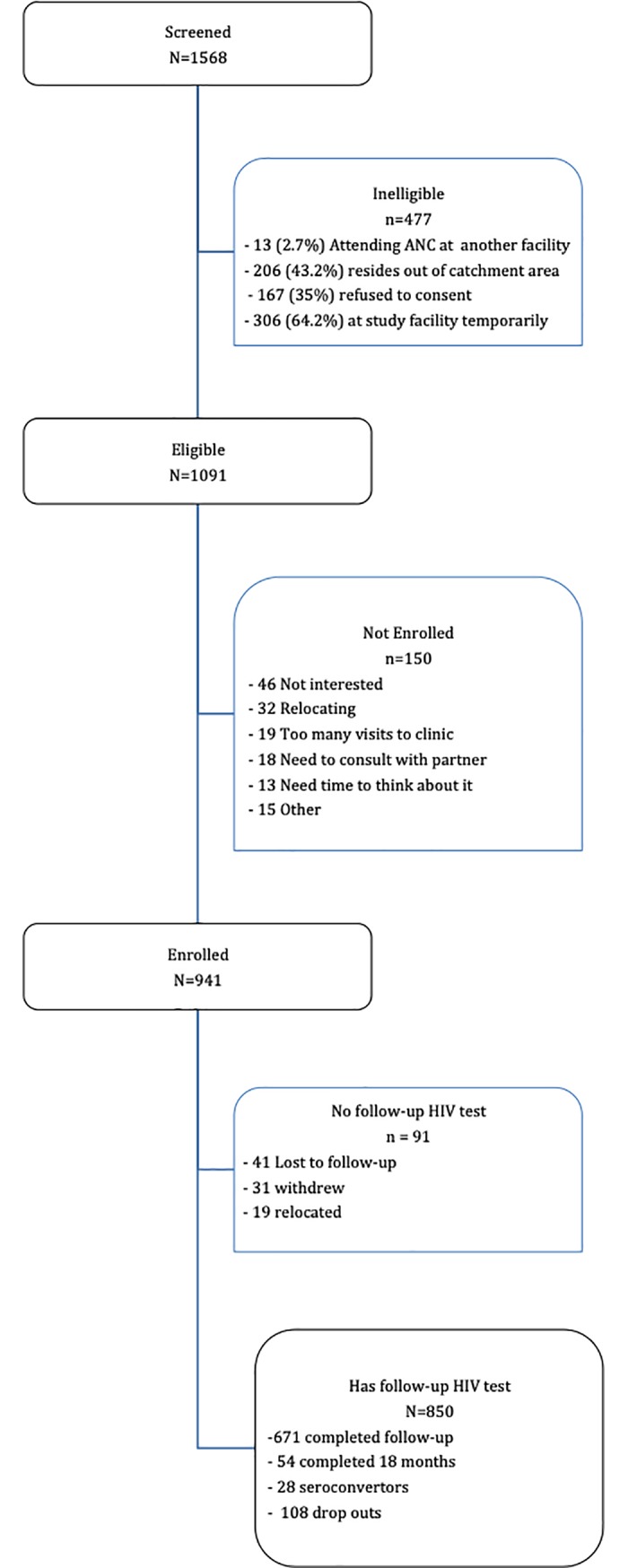
Study participant flow diagram.

Fig 1 presents the study screening, enrolment and follow-up numbers. Between September 2013 and August 2015, we enrolled 941 HIV-negative pregnant women from 13 health facilities in three districts of Lesotho (Table 1). Of these women, 850 (90.3%) had at least one follow-up HIV test after enrolment into the study and received 4224 HIV tests with a median of five HIV tests per woman. Loss to follow-up was significantly associated with district of residence; with more women from Mohale’s Hook being lost to follow up compared to Thaba Tseka and Buthe Buthe. Significantly more partners were tested and their test results known in women with at least one follow-up visit compared to women without follow-up visits. The distribution of other baseline characteristics did not differ between women with and women without follow-up.

**Table 1:** Baseline characteristics of all HIV negative women enrolled in study (n = 941) by presence of follow-up HIV test.

**Characteristic**	**Total**	**Follow-up** **N (%)**	**No follow-up** **N (%)**	**p-value**
**Number (total)**	941	850 (90.3)	91 (9.7)	
**District**				<0.001
Thaba Tseka Butha-Buthe Mohale’s Hook	288 380 273	277 (96.2) 346 (91.1) 227 (83.2)	11 (3.8) 34 (8.9) 46 (16.8)	
**Median Age (Min—Max)**	23 (14–45)	23 (14–45)	22 (14–41)	0.181
**Age group**				0.715
16–19 years 20–24 years 25–45 years	200 363 378	180 (90.0) 325 (89.5) 345 (91.3)	20 (10.0) 38 (10.5) 33 (8.7)	
**Marital Status**				0.083
Married Living with partner Never married Divorced / Widowed	801 4 128 8	731 (91.3) 3 (75.0) 109 (85.2) 7 (87.5)	70 (8.7) 1 (25.0) 19 (14.8) 1 (12.5)	
**Education**				0.260
None Primary Secondary Post-secondary	5 285 377 274	5 (100) 265 (93.0) 335 (88.9) 245 (89.4)	0 (0) 20 (7.0) 42 (11.1) 29 (10.6)	
**Partner’s age group**				0.126
< = 24 25–34 > = 35 Unknown	224 440 168 109	196 (87.5) 396 (90.0) 159 (94.6) 99 (90.8)	28 (12.5) 44 (10.0) 9 (5.4) 10 (9.2)	
**Partner’s education**				0.242
None Primary Secondary Post-secondary Unknown	50 366 199 269 57	48 (96.0) 335 (91.5) 180 (90.5) 239 (88.8) 48 (84.2)	2 (4.0) 31 (8.5) 19 (9.5) 30 (11.2) 9 (15.8)	
**Partner tested for HIV**				0.057
Yes No Don’t know	449 334 158	413 (92.0) 302 (90.4) 135 (85.4)	36 (8.0) 32 (9.6) 23 (14.6)	
**Partner’s HIV status**				0.051
Negative Positive Unknown	422 19 500	391 (92.7) 16 (84.2) 443 (88.6)	31 (7.3) 3 (15.8) 57 (11.4)	
**Sexually active in last 3 months**				0.357
Yes No	834 107	756 (90.7) 94 (87.9)	78 (9.3) 13 (12.1)	
**Condom use with main partner**				0.890
Always Most times Rarely Never	38 208 220 475	35 (92.1) 190 (91.4) 196 (89.1) 429 (90.3)	3 (7.9) 18 (8.6) 24 (10.9) 46 (9.7)	
**History of genital infection**				0.493
Yes No	213 728	195 (91.6) 655 (90.0)	18 (8.4) 73 (10.0)	
**Counseled on HIV prevention**				0.432
Yes No	736 205	667 (90.6) 183 (89.3)	69 (9.4) 22 (10.7)	

The women ranged between 14 and 45 years old with a median age of 23 years. Of the 941 women, 200 (21.2%) women were adolescents (<20 years). Most of the women (85.5%) were married or living with a partner, 13.6% had never married and 0.8% were either divorced or widowed. Partner HIV status was reported as known by 441 (46.9%) of women, with only 19 (4%) reporting an HIV-positive partner; the majority of women, 53.1%, did not know the HIV status of their partner. The women’s level of education was evenly distributed between post-secondary (29.1%), secondary (40.1%) and primary (30.3%) with only five (0.5%) women reporting no formal education. The median gestation at ANC booking was 27 weeks (range: 4–41 weeks). Approximately 28% of the women registered for ANC before 21 weeks, 37% between 21 and 28 weeks, and 35% registered after 28 weeks gestation. About half (50.5%) of the women reported never using condoms with their main partners.

Table 2 presents the estimated overall HIV incidence and period specific HIV incidences disaggregate by pregnancy and post-partum period. Twenty-eight (3.3%) of 850 women who tested HIV-negative at first ANC seroconverted during the study period. The 850 women contributed a total of 1777 person-years of follow up during pregnancy and postpartum period for an estimated overall HIV incidence rate of 1.58 (95% CI: 1.05–2.28) per 100 person years. On average (±SD), a woman was followed up for 25 ± 7 months.

**Table 2:** Overall, pre- and post-delivery HIV incidence by age group among women attending ANC in Lesotho.

**Period**		**No. of new HIV positives**	**N**	**Total person years follow-up**	**Incidence Risk [95% CI]**	**Incidence Rate [95% CI]** **(per 100 person years)**
**Overall**	All women	28	850	1777.0	3.3% [2.2–4.7]	1.58 [1.05–2.28]
	16–19 years	8	180	365.1	4.4% [1.9–8.6]	2.19 [0.95–4.32]
	20–24 years 25–45 years	15 5	325 345	663.6 748.3	4.6% [2.6–7.5] 1.4% [0.5–3.3]	2.26 [1.26–3.73] 0.67 [0.22–1.56]
**Pre-delivery**	All women	8	850	306.8	0.9% [0.4–1.8]	2.61 [1.12–5.14]
	16–19 years	2	180	66.1	1.1% [0.1–4.0]	3.02 [0.37–10.93]
	20–24 years 25–45 year	4 2	325 345	115.9 124.7	1.2% [0.3–3.1] 0.6% [0.1–2.1]	3.45 [0.94–8.83] 1.60 [0.19–5.79]
**Postpartum**	All women	20	842	1470.2	2.4% [1.4–3.6]	1.36 [0.83–2.10]
	16–19 years	6	178	299.0	3.4% [1.2–7.2]	2.01 [0.74–4.37]
	20–24 years 25–45 years	11 3	321 343	547.7 623.5	3.4% [1.7–6.0] 0.9% [0.2–2.5]	2.01 [1.00–3.59] 0.48 [0.10–1.41]

The median follow up time from first ANC to delivery was 4.4 months (range: 0.03–8.6). Out of 850 HIV-negative women, eight (0.9%) women had an estimated HIV seroconversion date before delivery. HIV incidence rate during pregnancy was estimated at 2.61 (95% CI: 1.12–5.14) per 100 person-years. HIV-negative women were followed up for a median period of 23.6 (range: 0.09–26) months post-delivery. During the postpartum follow-up, 20 (2.4%) out of the 842 HIV-negative women seroconverted, for an estimated HIV incidence rate of 1.36 (95% CI: 0.83–2.10) per 100 person-years. Although seroconversion rates were higher during pregnancy than postpartum, these differences were not statistically significant (IRR = 1.92, 95% CI: 0.73–4.54).

**Fig 2:**
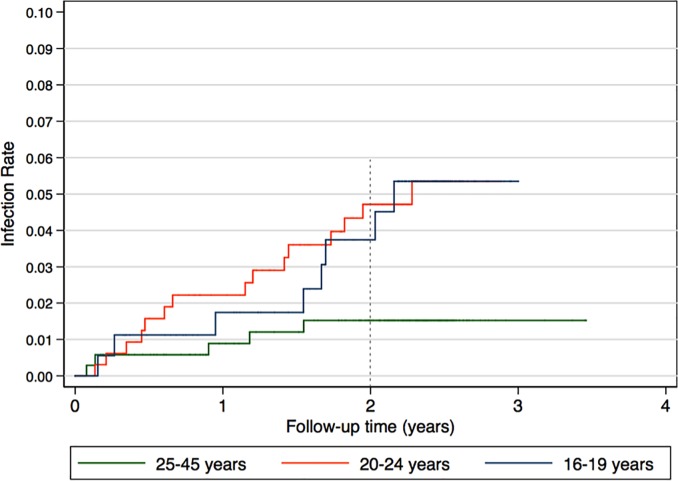
Time to HIV seroconversion by age group of women.

Among the 28 seroconverters, eight were between the ages of 16 and 19 years, 15 were aged 20 to 24 years and five were 25 years and older. Overall HIV incidence rate was significantly higher among adolescent (2.19 per 100 person years) and young women (2.26 per 100 person years) compared to adult women (0.67 per 100 person years). Fig 2 shows the distribution of time to seroconversion by age group. The hazard of HIV infection was significantly higher among adolescent and young women compared to adult women (rank sum p-value = 0.026).

Table 3 summarizes the socio-demographic and behavioral characteristics and their association with HIV seroconversion. Women 24 years old or younger had significantly higher risk of HIV infection compared to women older than 24 years (adjusted HR = 3.35, 95%CI: 1.16–9.69). Women presenting late (>20 weeks gestational age) for ANC had more than a six-fold increased hazard of HIV infection compared to women presenting early (< = 20 weeks gestational age) (adjusted HR = 6.36, 95%CI: 1.47–27.58). Women reporting more than one sexual partner since the last HIV negative test had a significantly higher hazard of HIV infection compared to women with one or no sexual partners during follow-up (adjusted HR = 3.38, 95% CI: [1.06–10.84]). Women reporting a urethral discharge during follow-up were significantly more likely to get infected with HIV compared to women not experiencing a urethral discharge (adjusted HR = 3.75, 95% CI: [1.42–9.87]. Women who did not take any preventive actions had a significantly increased hazard of HIV infection compared to women who practiced some preventive action including consistent condom use, asking partner’s HIV status, encouraging partners to get tested, committing to one sexual partner or abstaining from sex (adjusted HR = 3.53, 95% CI: [1.27–9.84]). Marital status, educational level, knowledge of sexual partner’s HIV status (negative or positive) at enrolment and repeat HIV test counseling were not independently associated with HIV acquisition.

**Table 3:** Number of HIV seroconversion by selected baseline characteristics and reported risk factors during follow-up among women attending ANC in Lesotho.

**Characteristic**	**Total (N)**	**New HIV n (%)**	**Unadjusted Hazard Ratio [95% CI]**	**Adjusted** **Hazard Ratio [95% CI]**	**p-value**
Baseline characteristics					
**Age group**					
25–45 years 14–24 years	345 505	5 (1.4) 23 (4.6)	- 2.91 [1.08–7.86]	- 3.35 [1.16–9.69]	- 0.026
**Marital Status**					
Married/live in Never married Divorced / Widowed	734 109 7	21 (2.9) 5 (4.6) 2 (28.6)	- 1.28 [0.41–3.95] 4.96 [0.65–37.71]	- 0.60 [0.18–2.01] 6.23 [0.75–51.95]	- 0.411 0.091
**Education**					
Primary / none Secondary Post-secondary	270 335 245	7 (2.6) 16 (4.8) 5 (2.0)	- 1.57 [0.62–3.98] 0.78 [0.24–2.60]		
**Gestational Age at 1**^**st**^ **ANC**					
< = 20 weeks >20 weeks	240 610	2 (0.8) 26 (4.3)	- 5.65 [1.34–23.85]	- 6.36 [1.47–27.58]	- 0.013
**Partner’s HIV status**					
Unknown Known HIV positive Known HIV negative	443 16 391	12 (2.7) 1 (6.2) 15 (3.8)	- 1.96 [0.25–15.25] 1.32 [0.61–2.83]		
**Behavioral Characteristics during follow-up**					
**Number of sexual partners**					
< = 1 >1	751 94	20 (2.7) 5 (5.3)	- 2.97 [0.98–8.99]	- 3.38 [1.06–10.84]	- 0.040
**Urethral discharge**					
No Yes	700 145	17 (2.4) 8 (5.5)	- 5.61 [2.20–14.29]	- 3.75 [1.42–9.87]	- 0.008
**Reported Preventive actions taken during follow-up**					
**Used condoms consistently**					
No Yes	206 639	10 (4.8) 15 (2.4)	- 0.42 [0.16–1.12]	- 0.86 [0.28–2.64]	- 0.791
**Asked partners’ HIV status**					
No Yes	637 208	20 (3.1) 5 (2.4)	- 1.66 [0.56–4.97]		
**Encouraged partners to get tested**					
No Yes	538 307	17 (3.2) 8 (2.6)	- 1.83 [0.67–5.00]		
**Committed to one partner**					
No Yes	245 600	14 (5.7) 11 (1.8)	- 0.27 [0.09–0.82]		
**Protective action taken**					
Some None	429 416	12 (2.8) 13 (3.1)	- 3.52 [1.51–8.23]	- 3.53 [1.27–9.84]	- 0.016
**Abstained from sex**					
No Yes	670 175	20 (3.0) 5 (2.9)	- 1.58 [0.36–6.81]		

Of the 28 HIV seroconverters, 23 (82%) were initiated on ART before study end: 19 (68%) were initiated on ART within a month of the initial HIV positive test, four (14%) were initiated after a month and five (18%) were not yet initiated on ART by study end. Among the 5 seroconverters not on ART, one declined while the rest were diagnosed at the study end visit.

Thirty infants; 26 singletons and two sets of twins were born to the 28 women with recent HIV infection. Of these, two were stillbirth and four others died while their mothers had not yet seroconverted. Of the 24 remaining, 21 (87.5%) were tested for HIV using either DNA PCR or antibody test during follow-up while three had not been tested for HIV by end of study. The estimated HIV transmission rate among children who were tested was 2/21 (9.5%, 95%CI: 1.2–30.4). Of note, the two children who tested HIV positive were diagnosed at the 24-month visit together with their mothers who seroconverted postpartum. One mother last tested HIV negative at 6 months postpartum and the other had a last HIV negative test at 18 months. Almost all infants (7/8) born to women who seroconverted before delivery were initiated on infant ARV prophylaxis immediately after delivery. However, among women who tested HIV positive after delivery and had a live birth (n = 16), 11 were diagnosed with HIV at the 24-month visit (study exit visit). None of the infants born to the 5 women who tested positive after delivery but before the 24-month visit were initiated on ARV prophylaxis.

## Discussion

We observed a 1.58% annual incidence rate of HIV infection among our cohort of HIV-negative women followed between first ANC visit and 24 months postpartum. Our results suggest that annual HIV incidence is higher during pregnancy (2.61%) compare to post-partum (1.36%). Our data also indicates that there is a significant risk of HIV transmission (9.5%) from acutely infected pregnant and postpartum women to their infants. Our results suggest there is a need to strengthen HIV prevention approaches during pregnancy and postpartum within this population including pre-exposure prophylaxis, regular HIV testing of pregnant and breastfeeding women as well their partners, and immediate initiation of infant ARV prophylaxis.

Our overall annual HIV incidence estimate was similar to the 2014 Lesotho Demographic Health Survey annual HIV incidence estimate among adult women of 1.7% (95% CI: 0.8–2.6). Although these estimates are high in an era of increased access to antiretroviral therapy, they are somewhat lower than reported in a meta-analysis, which included studies published between 1998 and 2012, reflecting an era when antiretroviral therapy for adults was either not available or relatively restricted. The majority of the women in our study did not know their partner’s HIV status and thus we are unable to know the proportion of women with HIV-positive sexual partners who might have been receiving antiretroviral therapy. Kinuthia et al. similarly found a high proportion of women who did not know their partner’s HIV status (33.6%) and a significant association with HIV acquisition[15]. During the time of the study (2013–2017), Lesotho introduced the ‘Test and Treat’ strategy in which antiretroviral therapy was recommended for all HIV-positive adults regardless of their CD4 count. However, with only 43% of men living with HIV on treatment in 2016[16], protecting women from HIV infection, especially during pregnancy and post-partum, still remains a challenge. Some women who reported an HIV negative partner status still seroconverted, highlighting the false sense of protection from HIV based on a partner’s status that can change if they have other sexual partners. While the Lesotho HIV testing guidelines recommend HIV testing every 3 months for breastfeeding women[10], our study data does not indicate compliance with these guidelines. These data suggest that all pregnant and breastfeeding HIV negative women should be considered for PrEP as a high-risk vulnerable population in order to protect them and the infant from infection. While the Lesotho National Treatment Guidelines recommend PrEP for HIV-negative individuals at significant risk of becoming infected with HIV[11], pregnant and breastfeeding women are not specifically targeted for PrEP unless if they are in a known discordant relationship, have multiple concurrent sexual partners, exchange money for sex or inject drugs. Given high proportions of women who do not know their sexual partners’ HIV status, screening based on known discordant relationships excludes a significant number of women at risk for infection. Pregnant and breastfeeding women should therefore be prioritized in the national guidelines.

Our findings are similar to a study in South Africa reporting on ‘success’ of combination prevention intervention[17][18]. Among 1356 women with a similar age distribution to our study participants, 11 new HIV infections were detected over 828.3 person-years of follow-up, with an annual HIV incidence rate of 1.33%. Similarly, in this South African study, although not statistically significant annual HIV incidence appeared higher during pregnancy compared to post-partum (1.49% versus 1.03%).

Adolescent and young women had a significantly higher risk of HIV infection compared to older women. We also found that younger women were less likely to protect themselves from HIV acquisition compared to older women. Our findings confirm several studies that have identified associations between younger women and lower condom use. Compared to older women, younger women often have less power to negotiate condom use, may have less control over the sexual relationship and have lower self-efficacy to avoid HIV infection[19][20]. These data support the need for targeting adolescent and young women within the ANC system and creating adolescent-friendly environments.

The estimated MTCT rate among women infected during pregnancy and postpartum was unacceptably high, similar to a South African study[21,22] although the precision around the estimate was low due to small numbers. A community household survey of 18–24 months old HIV exposed children in Lesotho had a somewhat lower MTCT rate at 5.7% (95% CI: 4.0–8.0) [23], highlighting the increased risk of HIV transmission among newly infected women. The time between the last negative HIV test and first positive test for the two women who infected their babies was 7 and 13 months. These data suggest that breastfeeding women at risk of HIV infection should be tested more frequently to avoid transmission to their babies[24].

The study was limited by the small number of HIV seroconversions resulting in low power to estimate mother to child transmission rates in newly infected women and imprecise estimates of risk factor effects. We also lack final infant infection status on 39% of their infant; however, in many infants (73%), infection in the mother was first documented at 24 months, and hence infant diagnostic testing may have occurred after data collection for this study closed. There was also limited information on male partner characteristics.

However, our study had several strengths including a well-characterized cohort of women with regular risk assessment and HIV testing throughout pregnancy and postpartum, low attrition rates and geographic representation across the four ecological zones in Lesotho.

Our findings highlight the antepartum and breastfeeding periods as periods of increased risk for HIV acquisition by women, particularly for adolescent and young women. Our results suggest there is an increased need for counseling and support of HIV-uninfected pregnant and breastfeeding women to stay HIV-negative, including provision of pre-exposure prophylaxis (PrEP) during this high-risk period[25]. Additionally, our results demonstrate the critical need to provide repeat HIV testing during pregnancy and breastfeeding for women in high prevalence settings to enable early detection of HIV infection and initiation of ART, both for maternal health and to prevent transmission of HIV to their infants. Improving male partner HIV testing to identify infected male partners and ensuring they initiate ART, for their own health as well as to protect their female partner and their child, is also a critical component to HIV prevention for pregnant and breastfeeding women.

## Supporting information

S1 File Data on time to HIV acquisition, socio-demographic and behavioral factors. (XLSX)

S2 File Longitudinal data on HIV acquisition and behavioral factors. (XLSX)
